# Quantitative analysis of peripapillary RNFL capillary density and thickness in patients with mild nonproliferative diabetic retinopathy

**DOI:** 10.3389/fendo.2024.1404157

**Published:** 2025-01-24

**Authors:** Zelie Cao, Tao Tian, Ru Liu, Jinli Peng, Guoping Kuang

**Affiliations:** ^1^ Department of Ophthalmology, The Affiliated Chenzhou Hospital, Hengyang Medical School, University of South China, Chenzhou, Hunan, China; ^2^ The Aier Eye Hospital Affiliated with Jinan University, Shenzhen, China

**Keywords:** diabetic retinopathy, optical coherence tomography angiography, optic disc, vascular density of the peripapillary radial capillary plexus, retinal nerve fiber layer

## Abstract

**Purpose:**

Optical coherence tomography angiography was used to compare the changes of capillary density and thickness of the peripapillary retinal nerve fiber layer in patients with diabetes mellitus (DM) without diabetic retinopathy (DR) and patients with mild nonproliferative diabetic retinopathy (NPDR).

**Methods:**

In this prospective cross-sectional study, 62, 85, and 75 eyes of normal control group (NC), DM group, and NPDR group were included, respectively. All subjects received an optic-disc-centered 6 × 6-mm fundus scan. The annular region outside the optic disc boundary was divided into eight sectors: nasal superior, nasal inferior, inferior nasal (IN), inferior temporal (IT), temporal superior, temporal inferior, superior nasal, and superior temporal (ST). The average retinal nerve fiber layer thickness, average capillary density, and changes in thickness and capillary density of each sector in the annular region were calculated.

**Results:**

Compared with the NC group, the average capillary density of pRNFL in patients with DM and NPDR groups decreased significantly (*p* < 0.001), and there were significant differences in capillary density in all sectors except the IN sector in the DM group. There was no significant difference in the average thickness of pRNFL between the groups. Only the thickness of ST and IT in DM group and NPDR group, respectively, were significantly lower than that in the control group. In addition, the ROC curve of pRNFL average capillary density has high sensitivity and specificity in distinguishing DR from healthy eyes (AUC = 0.852).

**Conclusion:**

The pRNFL average capillary density in diabetic patients without DR and patients with mild NPDR, respectively, were significantly lower than that in healthy controls, especially in the NPDR group.

## Introduction

Diabetic retinopathy (DR) is one of the most serious ocular complications of diabetes and a serious blinding eye disease (1–3). According to the World Diabetes Federation, the total number of patients with diabetes worldwide was 536.6 million in 2022, and it is estimated that it will reach 783 million in 2045. Early DR typically does not cause symptomatic visual loss, and while chronic hyperglycemia is the main risk factor for visual loss due to DM, it does not always result in visual loss. The existence of proliferative diabetic retinopathy (PDR) at the first visit is usually related to delayed DM diagnosis or inadequate screening protocols.

In order to evaluate the degree of fundus lesions in patients with DR, ophthalmoscopy, fundus photography, optical coherence tomography (OCT), and fundus fluorescein angiography (FFA) are usually used. If the changes of retinal microcirculation can be found and intervened in time in the early stage, the visual quality prognosis of DR may be improved.

In recent years, optical coherence tomography angiography (OCTA) has provided a new tool for the early screening of DR. OCTA has been shown to be more sensitive than a doctor’s examination or fundus imaging, and capillary loss is detected earlier when the clinical lesions of DR become apparent (4–7). OCTA utilizes the contrast of light reflectance between blood and tissue to extract the blood flow signal and generate the image of vascular structure. It has a built-in quantitative analysis index to measure vascular morphology and density, which can provide detailed retinal blood flow and thickness information (8). In the past, most studies were limited to the early changes of macular blood flow density and FAZ in patients with DR or DR at different stages, while there were few studies on the changes of optic disc area in patients with early DR (9, 10). The purpose of this study was to compare the changes of capillary density and thickness of pRNFL within early DR, DM, and healthy individuals in order to achieve the purpose of early diagnosis of NPRD so as to provide guidance for clinical practice.

## Materials and methods

### Participants

This was a prospective cross-sectional study. This study recruited 113 subjects who came to the Department of Ophthalmology or Endocrinology of Chenzhou Hospital affiliated to University of South China from August to December 2023 and conformed to the tenet of the Declaration of Helsinki and was approved by the hospital’s ethics committee (no. 2023144). Written informed consent was provided by all patients before joining the study.

Inclusion criteria: (1) diagnosed with type 2 DM (DM was diagnosed according to the criteria set by the American Diabetes Association (11)), (2) older than 18 years, and (3) agrees to and cooperates with relevant ophthalmic examinations.

Exclusion criteria: (1) optical media opacities, myopia with spherical equivalent < -1.50 DS, (2) glaucoma or binocular IOP difference greater than 5 mmHg, (3) has a history of ocular trauma or has received fundus treatment, such as retinal laser photocoagulation, intravitreal injection, fundus retinal surgery, etc., (4) nystagmus, strabismus, amblyopia, and other eye gaze function is poor, (5) has a history of serious hypertension, heart disease, blood system diseases, and other diseases affecting the blood flow of the optic disc, and (6) there are age-related macular degeneration, uveitis, retinal detachment, and other fundus diseases other than DR.

### Demographic data and ophthalmic examination

Data available for all patients included patient characteristics, duration of diabetes, type of diabetes, and treatment. The ocular examination performed included best corrected visual acuity (BCVA), intraocular pressure (IOP), computerized optometry, OCT, OCTA, and fundus photography. All patients included in the DM and NPDR groups completed laboratory tests including fasting blood glucose (FBG), glycated hemoglobin A1c (HbA1c), and fasting C-peptide. After mydriasis, fundus examination was performed by two experienced ophthalmologists. The subjects were divided into three groups: normal control (NC), diabetes mellitus without DR (DM), and mild nonproliferative diabetic retinopathy (NPDR).

### OCTA examination

**Figure 1:**
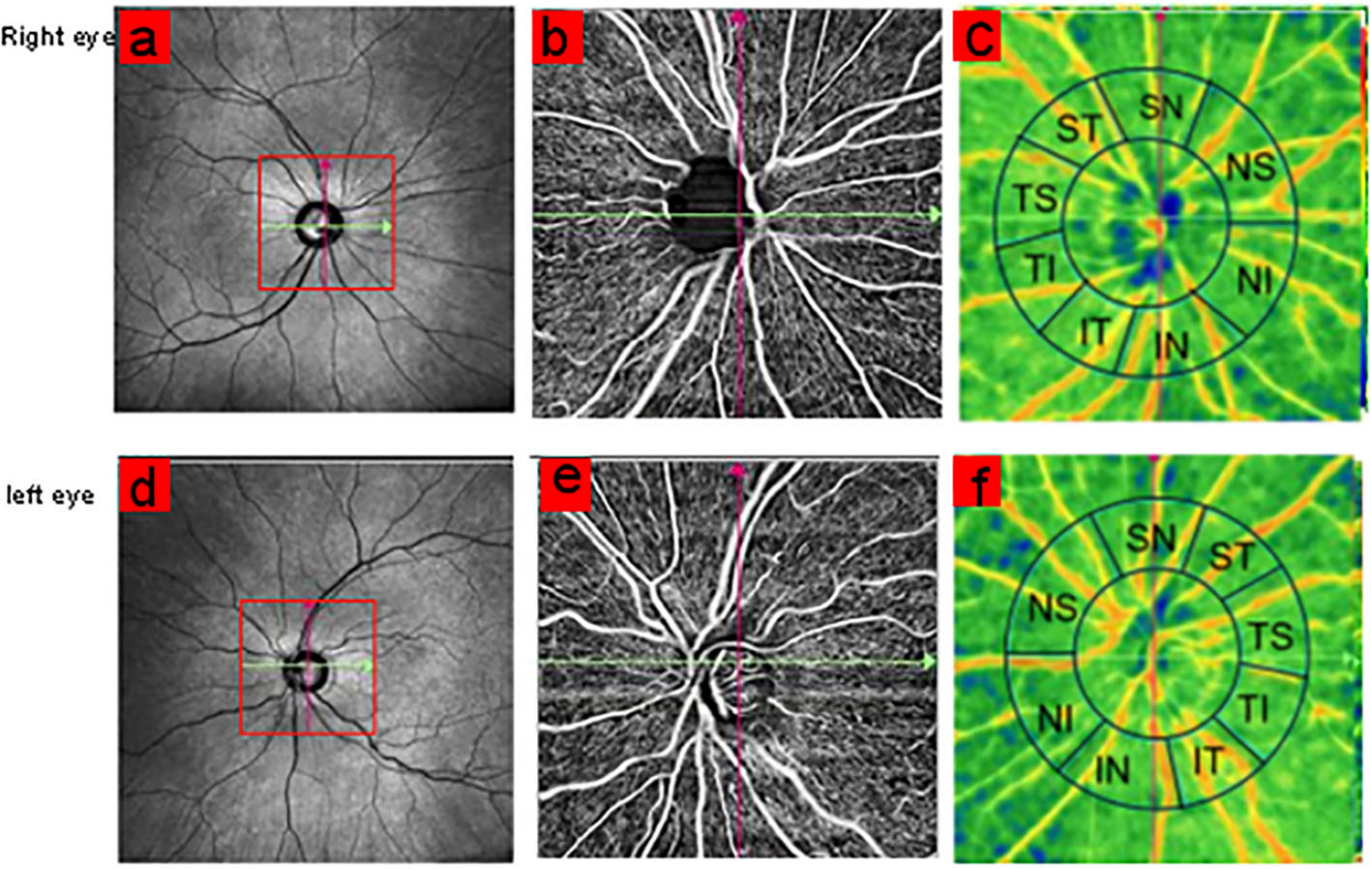
Scan centered on optic disc with 6 × 6 mm size area **(A, D)**. The annular region outside the optic disc boundary was divided into eight sectors: NS, NI, IN, IT, TS, TI, ST, and SN **(C, F)**.

The patients were examined using TowardPi high-speed octa system (YG100k Yalkaid, TowardPi Medical Technology Co., Ltd., Beijing, China). The device is equipped with a swept-source vertical-cavity surface-emitting-laser (VCSEL) with a wavelength of 1,060 nm and has a scanning rate of up to 400,000 A/scans per second. The scan area was 6.0 mm × 6.0 mm (centered on the optic disc) (**Figure 1**). The boundary of the optic disc is automatically determined by the computer system. The RNFL of the annular area 1 mm wide outside the optic disc boundary is defined as the peripapillary retinal nerve fiber layer (pRNFL) and is divided into eight sectors: nasal superior (NS), nasal inferior (NI), inferior nasal (IN), inferior temporal (IT), temporal superior (TS), temporal inferior (TI), superior temporal (ST), and superior nasal (SN). The RNFL blood flow density and RNFL thickness of each sector are independently analyzed and calculated by the system’s built-in software. Each eye was measured at least twice, and the images were graded 1–10 by OCTA platform. The scanned images with quality less than 8 were excluded, and the image with the highest quality was selected for data analysis.

### Statistical analysis

All statistical analyses in this study were performed using SPSS software (version 25.0 SPSS, Inc, Chicago, IL, USA). The Kolmogorov–Smirnov test is used for normality test, and normally distributed variables are expressed as mean and standard deviation (SD). Nonparametric variables were expressed as median and interquartile range (IQR). Finally, the differences between groups were evaluated by ANOVA or Kruskal–Wallis test with Bonferroni correction, respectively. A *p*-value <0.05 was considered statistically significant. Receiver operating characteristic (ROC) analysis was used to determine the effect of OCTA index in diagnosing early DR, and the curve was drawn and the area under the curve (AUC) was calculated.

## Results

### Baseline data

The general characteristics of the 113 patients are shown in **Table 1**. There were no significant differences in gender composition, age, IOP, and fasting C-peptide among the three groups. There were significant differences in blood glucose (*p =* 0.005) and HbA1c (*p* = 0.006) between DM and NPDR groups (**Table 1**).

**Table 1:** Basic characteristics of the 113 patients.

	NC	DM	NPDR	*p*-values
Number of eyes, *n*	62	85	75	–
Gender, male/female	13/18	27/17	24/14	*χ* ^2^ = 0.3768, *p* = 0.151
Age, year	49.90 ± 13.83	51.22 ± 11.08	53.07 ± 12.27	*F* = 0.587, *p* = 0.558
IOP, mmHg	15.90 ± 2.46	15.58 ± 2.55	15.49 ± 2.65	*F* = 0.467, *p* = 0.628
FBG, mmol/L	–	7.19 (5.91, 9.42)	10.61 (6.96, 15.29)	*U* = 534.000, *p* = 0.005
HbAlc, %	–	7.15 (6.40, 9.07)	9.60 (7.07, 11.27)	*U* = 539.000, *p* = 0.006
FCP, ng/mL	–	1.65 (1.44, 2.39)	1.81 (1.32, 2.76)	*U* = 769.500, *p* = 0.536

*p* < 0.05 indicates a statistically difference.

NC, normal control; DM, diabetes mellitus; NPDR, nonproliferative diabetic retinopathy; IOP, intraocular pressure; FBG, fasting blood glucose; HbA1c, glycosylated hemoglobin type A1c; FCP, fasting C-peptide; *χ* ^2^, chi-square test; *F*, one-way ANOVA; *U*, Mann–Whitney *U*-test; ±, mean ± standard deviations; (), median (interquartile ranges).

### Capillary density and thickness analysis of eight sectors

The thickness and blood flow density parameter values of the eight areas around the disc in each group are shown in **Table 1**. Compared with the control group, RNFL thickness was significantly different in the ST, SN, and IT sectors in the DM group (*p* < 0.05) and significantly different in the ST, IN, and IT sectors in the NPDR group (*p* < 0.05). Comparing between DM and NPDR groups, only the TI, NI and TS regions showed a significant increase in thickness, and the blood flow density in the ST and IN regions significantly decreased. Compared with the blood flow density of the eight sectors in the NC group, the blood flow density in all seven areas, except the IN area in the DM group, was significantly decreased (*p* < 0.05), and the blood flow density in all areas in the NPDR group was significantly decreased (*p* < 0.05) (**Table 2**).

**Table 2:** pRNFL blood flow density and thickness analysis of eight sectors in NC, DM, and NPDR.

Sector	NC	DM	NPDR	p1	p2	p3
pRNFL thickness (hic						
TS	71.00 (67.00, 81.000)	70.00 (66.00, 78.000)	75.00 (68.00, 88.000)	0.564	0.054	0.012
ST	137.00 (127.00, 151.25)	127.00 (113.50, 142.00)	127.00 (115.00, 143.00)	0.002	0.004	0.812
SN	125.50 (112.75, 145.25)	140.00 (119.00, 153.00)	133.00 (114.00, 151.00)	0.012	0.160	0.267
NS	90.50 (81.75, 100.25)	95.00 (86.50, 102.50)	96.00 (87.00, 105.00)	0.231	0.076	0.487
NI	71.00 (67.00, 80.250)	72.00 (65.50, 79.500)	75.00 (69.00, 84.000)	0.615	0.148	0.033
IN	129.50 (119.75, 139.75)	129.00 (112.50, 142.00)	123.00 (106.00, 141.00)	0.622	0.048	0.238
IT	157.50 (146.00, 168.25)	145.00 (131.00, 160.00)	144.00 (131.00, 160.00)	0.001	0.001	0.843
TI	79.50 (73.00, 84.25)	77.00 (71.50, 83.00)	80.00 (72.00, 91.00)	0.122	0.473	0.047
pRNFL blood flow density (%)						
TS	46.00 (45.00, 47.00)	45.00 (43.00, 46.00)	45.00 (43.00, 46.00)	0.001	0.001	0.566
ST	50.00 (48.00, 52.00)	48.00 (46.00, 50.00)	47.00 (45.00, 49.00)	0.000	0.000	0.029
SN	50.00 (48.00, 52.00)	49.00 (47.00, 51.00)	48.00 (47.00, 50.00)	0.029	0.001	0.156
NS	48.00 (46.00, 49.00)	46.00 (44.00, 49.00)	47.00 (44.00, 48.00)	0.001	0.001	0.835
NI	46.00 (44.00, 47.00)	44.00 (42.00, 46.00)	44.00 (40.00, 46.00)	0.001	0.000	0.257
IN	50.00 (48.00, 51.00)	50.00 (48.00, 51.00)	48.00 (46.00, 50.00)	0.157	0.000	0.005
IT	51.00 (50.00, 53.00)	50.00 (48.00, 52.00)	50.00 (47.00, 51.00)	0.001	0.000	0.051
TI	47.00 (45.00, 48.00)	46.00 (44.00, 47.00)	45.00 (43.00, 47.00)	0.008	0.004	0.451

pRNFL, the peripapillary retinal nerve fiber layer; NC, normal control; DM, diabetes mellitus; NPDR, nonproliferative diabetic retinopathy; TS, temporal superior; ST, superior temporal; SN, superior nasal; NS, nasal superior; NI, nasal inferior; IN, inferior nasal; IT, inferior temporal; TI, temporal inferior; p1, NC vs. DW; p2, NC vs. NPDR; p3, DM vs. NPDR; (), median (interquartile ranges).

### pRNFL average capillary density and thickness analysis of eight sectors

**Figure 2:**
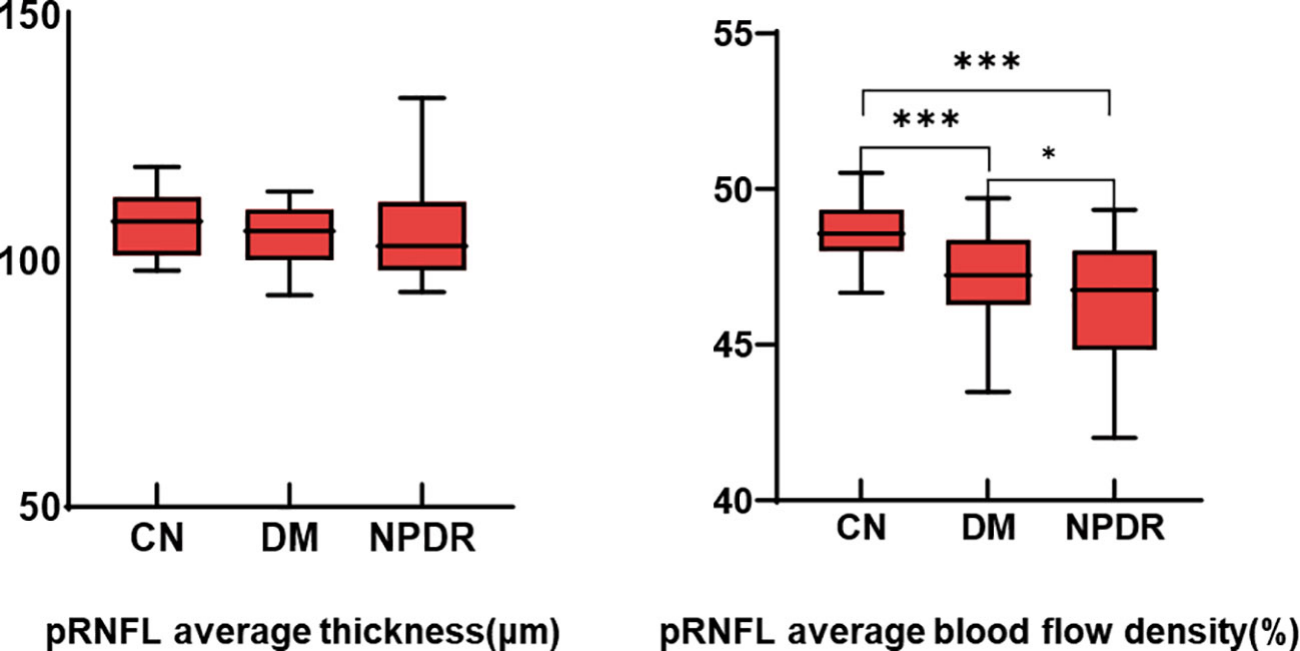
Comparison of pRNFL average thickness and average blood flow density. *:p<0.05, ***:*p*<0.001.

The mean RNFL thickness was not statistically different among the three groups. Compared with the NC group, the average blood flow in the DM and NPDR groups decreased significantly (*p* < 0.001), and there was also a statistical difference between the DM and NPDR groups (*p* < 0.05) (**Table 3** and **Figure 2**).

**Table 3:** pRNFL average blood flow density and thickness analysis of eight sectors.

Sector	NC	DM	NPDR	p1	p2	p3
pRNFL average thickness (μm)	108.00 (101.00, 113.00)	106.00 (100.00, 110.50)	103.00 (98.00, 112.00)	0.313	0.146	0.466
pRNFL average blood flow density (%)	48.56 (48.00, 49.34)	47.23 (46.28, 48.37)	46.76 (44.83, 48.03)	0.000	0.000	0.018

pRNFL, peripapillary retinal nerve fiber layer; NC, normal control; DM, diabetes mellitus; NPDR, nonproliferative diabetic retinopathy; p1, NC vs. DW; p2, NC vs. NPDR; p3, DM vs. NPDR.

### ROC curves in the NC and DR groups

**Figure 3:**
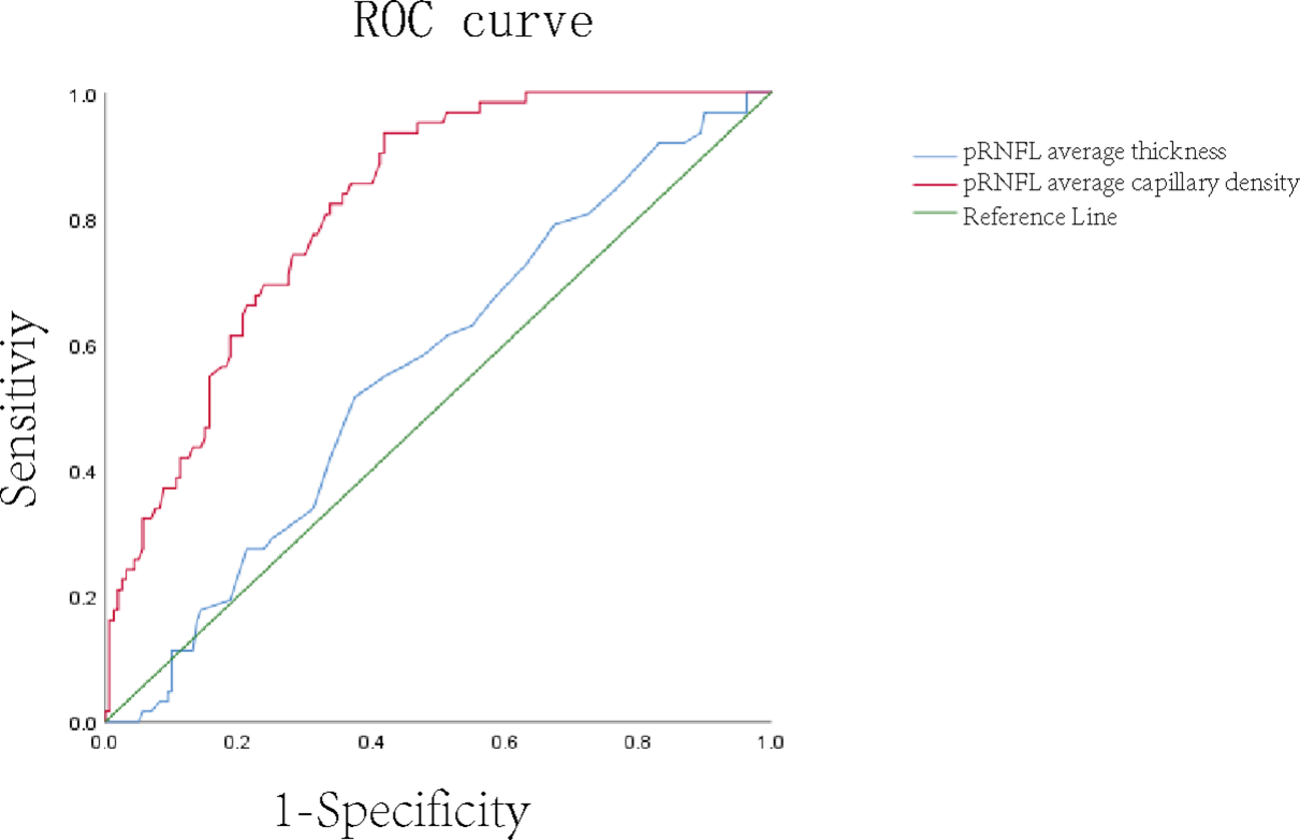
ROC curve of average thickness and average blood flow density of pRNFL.

The ROC curve analysis with an AUC of 0.852 showed the ability of pRNFL average capillary density to distinguish normal eyes from DR eyes, while the AUC of pRNFL average thickness was only 0.572 (**Figure 3**).

## Discussion

In the past, the research on early DR patients mainly focused on the fundus macular area and peripheral retinal and choroidal thickness. Previous studies have shown that the FAZ area of the retinal macula in patients with early DR is expanded (12), the retinal capillary density is decreased (13–15), and the choroidal vascular index (CVI) and choroidal thickness (CHT) are also significantly reduced (16). However, there are few studies on the early changes of the optic disc in DR patients. In this study, an optic-disc-centered, 6 × 6-mm area scan was performed, revealing the changes of pRNFL blood flow and thickness in early DR. Compared with the control group, the average blood flow density in each sector or annular area in the DM group and the NPDR group decreased significantly. The ROC curve analysis showed the predictive value of pRNFL average blood flow density for early DR, but the diagnostic significance of average thickness was not significant. It shows that pRNFL mean blood flow density is a very meaningful biomarker, which provides ideas for the development of new diagnostic and follow-up methods. In diabetic patients, hyperglycemia will produce free radicals and advanced glycation end products by triggering metabolic pathways such as polyol and hexosamine pathways, leading to inflammation and ischemia (17, 18). The nutrition of pRNFL is supplied by a unique capillary plexus (19, 20), which mainly comes from the branches of the central retinal artery (21, 22), and the high energy demand for unmyelinated axons located in pRNFL makes them extremely vulnerable to ischemic injury (23–25). The decrease of pRNFL blood flow density can be observed in the early stage of DR, which is confirmed by our results, and is similar to the research of Zhang et al. (26). Previous studies have shown that the RNFL thickness is higher in the inferior sector, followed by the superior and nasal sector, and the thinnest in the temporal sector (27, 28), and there is a positive correlation between pRNFL blood flow density and RNFL thickness (29, 30). It is also positively correlated with each sector (31), so it is expected that the density of retinal ganglion cells (RPCs) and the RNFL thickness distribution are similar. Li et al. (32) and others also showed that pRNFL thickness became thinner in the early stage of DR, but this was different from our results. Although the average thickness among the three groups showed a downward trend, it did not show significant statistical differences. We only observed that the thickness of ST and IT in the DM group and the NPDR group was significantly lower than that in the NC group. RPCs located in the superior or inferior temporal maculae send their axons in an arcuate manner to the superior temporal and inferior temporal portions of the optic nerve, respectively (33), making these locations abnormal earlier than the other regions in the early stage of the disease. RNFL thinning at the edge of the optic disc in the early stage of glaucoma usually also occurs first in the superior temporal and inferior temporal regions (34).

There are still some limitations in this study. Firstly, the sample size of this study is relatively small, which may affect the reliability and generalizability of the results. Future studies can expand the sample size to increase the reliability of the results. Secondly, this study adopts a cross-sectional design, and further research can adopt a longitudinal tracking design with long-term follow-up to observe the blood flow density and thickness of pRNFL so as to better observe its relationship with the development of DR. Moreover, we cannot explore the relationship between the changes of blood flow density and thickness around the optic disc and the risk of cardiovascular disease and the level of diabetes control in diabetic patients. Future studies can further study these related factors and comprehensively analyze their predictive value for retinopathy. A further study on these related factors and validation of the utility of these parameters as biomarkers of diabetic retinopathy are of great significance for the diagnosis and treatment of DR.

In conclusion, the results of this study showed that pRNFL capillary density was significantly changed in patients with early DR. There was no significant difference in the average thickness of pRNFL between the groups. Only the thickness of ST and IT, respectively, in the DW group and the NPDR group were significantly lower than those in the control group. These findings suggest that there are changes in the blood vessels and structure of the optic disc in the early stage, which may have important significance for the early detection of DR. It also confirms that the average blood flow density of pRNFL is a very meaningful biological parameter, which provides ideas for the development of new diagnostic methods and follow-up methods for mild nonproliferative diabetic retinopathy.

## Data Availability

The raw data supporting the conclusions of this article will be made available by the authors, without undue reservation.
